# Identifying Mn^VII^-oxo Species during Electrochemical Water Oxidation by Manganese Oxide

**DOI:** 10.1016/j.isci.2018.05.018

**Published:** 2018-05-30

**Authors:** Biaobiao Zhang, Quentin Daniel, Lizhou Fan, Tianqi Liu, Qijun Meng, Licheng Sun

**Affiliations:** 1 Department of Chemistry, KTH Royal Institute of Technology, Stockholm 10044, Sweden; 2 State Key Laboratory of Fine Chemicals, Institute of Artificial Photosynthesis, DUT-KTH Joint Education and Research Center on Molecular Devices, Dalian University of Technology (DUT), Dalian 116024, P. R. China

**Keywords:** Inorganic Chemistry, Surface Science, Energy Materials, Electrocatalysis

## Abstract

Identifying surface active intermediate species is essential to reveal the catalytic mechanism of water oxidation by metal-oxides-based catalysts and to develop more efficient catalysts for oxygen-oxygen bond formation. Here we report, through electrochemical methods and *ex situ* infrared spectroscopy, the identification of a Mn^VII^ = O intermediate during catalytic water oxidation by a *c*-disordered *δ*-MnO_*x*_ with an onset-potential-dependent reduction peak at 0.93 V and an infrared peak at 912 cm^−1^. This intermediate is proved to be highly reactive and much more oxidative than permanganate ion. Therefore, we propose a new catalytic mechanism for water oxidation catalyzed by Mn oxides, with involvement of the Mn^VII^ = O intermediate in a resting state and the Mn^IV^−O−Mn^VII^ = O as a real active species for oxygen-oxygen bond formation.

## Highlights

• A reactive Mn^VII^-oxo intermediate was identified during water oxidation by a MnO_*x*_
• The Mn^VII^-oxo species was proved to be much more oxidative than permanganate ion
• The Mn^IV^−O−Mn^VII^ = O moiety is a real highly active state for O–O bond formation
• A new mechanism for Mn oxide-catalyzed electrocatalytic water oxidation is proposed

## Graphical Abstract

**Figure:**
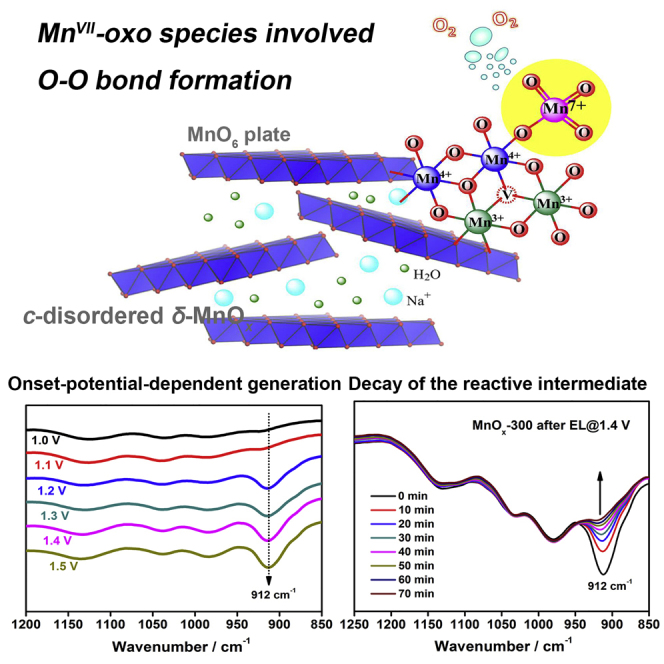

## Introduction

The continuous extraction of electrons and protons from water is a key step in sustaining life on Earth, and research on such processes is crucial for developing renewable energy systems via artificial photosynthesis (Walter et al., 2010). In nature, water oxidation occurs at the Mn_4_CaO_5_ cluster in photosystem II (PSII), with a low overpotential of around 160 mV and high rate of 100–400 s^−1^(Cox et al., 2013, Najafpour et al., 2016, Umena et al., 2011, Limburg et al., 1999). Although Mn complexes and oxides have been developed as promising water-oxidation catalysts (WOCs) (Najafpour et al., 2016, Limburg et al., 1999), the activity gaps between artificial catalysts and the Mn_4_CaO_5_ cluster are large (Najafpour et al., 2016). One primary reason for the ineffectual development of Mn-based WOCs is our limited understanding of the water-oxidation mechanism in PSII. In conventional mechanistic models, Mn^IV^ = O^⋅^ and Mn^V^ = O species are widely accepted as key intermediates for both Mn_4_CaO_5_ clusters and synthetic catalysts (Cox et al., 2013, Zahran et al., 2016, Najafpour et al., 2016, Jin et al., 2017). However, these mechanisms do not reflect the unique redox chemistry of Mn (five valences, varying from Mn^II^ to Mn^VII^, incorporation of many disproportionation and comproportionation reactions, moderate oxidation potentials from Mn^II^ to Mn^VII^) and the fact that low-valent Mn^III^ species and high-valent MnO_4_^−^ species are usually observed during water-oxidation catalysis (Takashima et al., 2012, Yagi and Narita, 2004, Limburg et al., 1997, Limburg et al., 1999). Significant improvements in the catalytic performance of Mn-based WOCs could be aided by the exploration of more appropriate catalytic mechanisms for Mn-based catalysts. Here, we report the first direct experimental evidence for the formation of a Mn^VII^ = O intermediate during catalytic water oxidation on a *c*-disordered *δ*-MnO_*x*_, MnO_*x*_-300, which was previously developed by our group (Zhang et al., 2017). On the basis of this discovery, an innovative water-oxidation mechanism that involves Mn^VII^ = O is proposed. This new information on water oxidation with a Mn-based catalyst might help designing more efficient Mn-based WOCs for artificial photosynthesis.

## Results

### Observation of an Intermediate State at 0.93 V by Electrochemical Study

**Figure 1:**
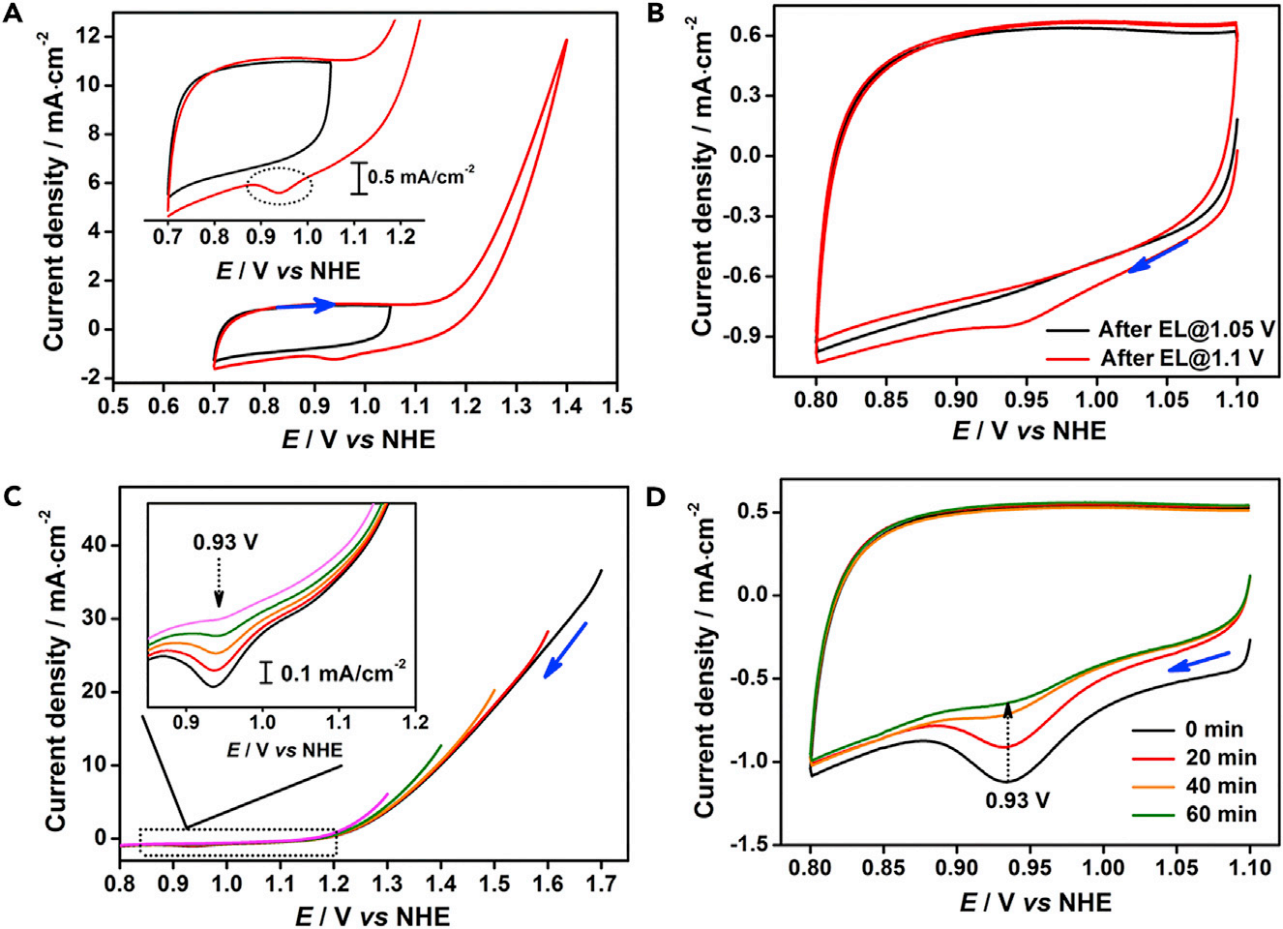
Observation of Intermediate State at 0.93 V vs Normal Hydrogen Electrode (NHE) by Cyclic Voltammetry (CV) (A) CV curves of MnO_*x*_-300 in two different potential regions. The inset shows enlarged CV curves. (B) Negative-scan CV curves of MnO_*x*_-300 after electrolysis at 1.05 V and 1.1 V for 10 min (C) Negative-scan linear sweep voltammetry (LSV) curves swept from 1.7 V (black curve), 1.6 V (red curve), 1.5 V (orange curve), 1.4 V (green curve), and 1.3 V (magenta curve). The inset shows enlarged parts of the LSV curves. (D) Negative-scan CV curves of MnO_*x*_-300 after electrolysis at 1.4 V for 2 min with different delay times. The MnO_*x*_-300 electrode was removed after electrolysis, quickly rinsed with water, and dried in a N_2_ flow. It was then kept under air for 20, 40, and 60 min, respectively, before the CV was recorded. Electrolyte was 1.0 M KPi (pH 7). Scan rate was 10 mV/s. Blue arrows show scan direction. See also Figures S1–S4.

The cyclic voltammetry (CV) curve of MnO_*x*_-300 (Figure 1A) shows that as the potential increases, the MnO_*x*_-300 film is gradually oxidized. When the potential approaches the water-oxidation onset potential, at around 1.1 V (Zhang et al., 2017), the active Mn sites are oxidized to a high oxidation state, followed by the evolution of oxygen. There are no distinct oxidation peaks, and only one broad wave is observed before the initiation of water oxidation at around 1.1 V versus the normal hydrogen electrode (NHE), indicating the presence of strong electronic interactions between Mn sites in MnO_*x*_-300 (Zaharieva et al., 2012). This high oxidation state of MnO_*x*_-300, which does not give distinct oxidation peaks, is completely unexpected. High oxidation states, such as Mn^IV^, Mn^V^, Mn^VI^, and Mn^VII^ species, may be possible intermediates involved in this state.

However, we observed a distinct reduction peak at 0.93 V during a negative scan; this peak does not appear when the potential is swept in the range 0.7–1.05 V, in which water oxidation does not occur (Figure 1A). In addition, there is no evident reduction peak in the potential region 0.7–1.5 V in the CV curve of MnO_*x*_-300 in a CH_2_Cl_2_ electrolyte, in which no water is present (Figure S1). Figure 1B shows that the peak at 0.93 V appears in the first cycle in the CV curve of MnO_*x*_-300 after electrolysis (EL) at 1.1 V, at which water oxidation is initiated (Zhang et al., 2017). The reduction peak vanishes in the second cycle because the relevant species were reduced during the first scan. In contrast, no reduction peak is observed for MnO_*x*_-300 after electrolysis at 1.05 V. The negative-scan linear sweep voltammetry (LSV) curves in Figure 1C show that the increment in the current intensity of the peak at 0.93 V is consistent with the increase in the catalytic current density with increasing LSV initial potential.

These results clearly show that the reduction peak at 0.93 V is attributable to the reduction of an active intermediate state, and its generation strictly corresponds to initiation of the water-oxidation reaction. We further found that the intermediate state rapidly and repeatedly accumulates in MnO_*x*_-300 after electrolysis at 1.4 V, at which voltage catalytic water oxidation is fast (Figures S2–S4). Figure 1D shows that complete degradation of the generated active intermediate state occurred in a period of 60 min; this can be considered as its lifetime under the experimental conditions.

### Observation of the Intermediate State at 912 cm^−1^ by Infrared Spectroscopy

**Figure 2:**
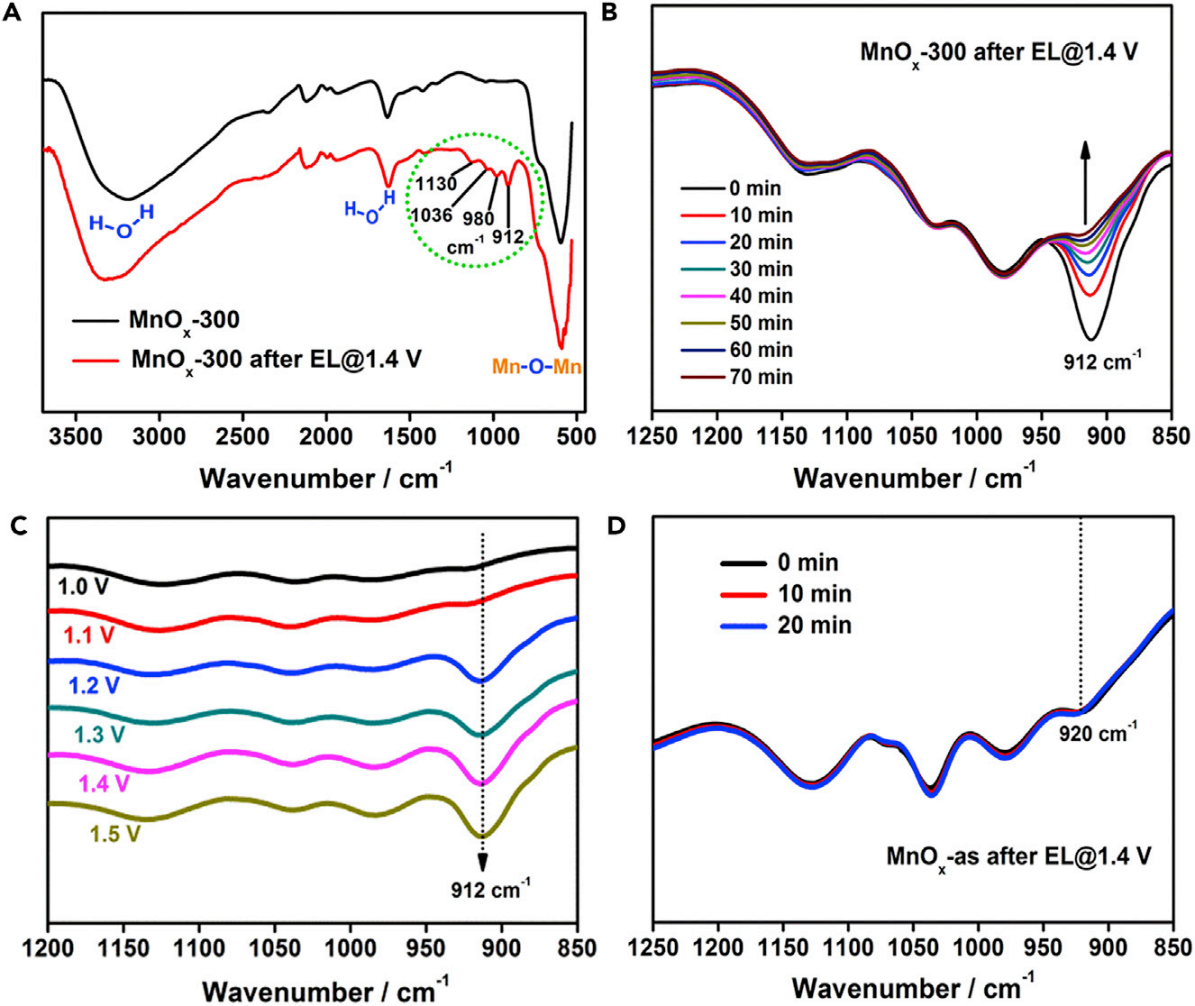
Observation of Intermediate State at 912 cm^−1^ by IR Spectroscopy (A) IR spectra of pristine MnO_*x*_-300 and MnO_*x*_-300 after electrolysis at 1.4 V. (B) Time-resolved IR spectra of MnO_*x*_-300 after electrolysis at 1.4 V. During the aging period, the sample was just kept on the sample holder without any other operation. (C) IR spectra of MnO_*x*_-300 after electrolysis at different potentials. (D) Time-resolved IR spectra of MnO_*x*_-as after electrolysis at 1.4 V. See also Figures S5–S7.

The lifetime of the observed intermediate state is on a 1 hr timescale; therefore it is possible to identify the structure of the intermediate by *ex situ* attenuated total reflection Fourier transform infrared (ATR-FTIR) spectroscopy, which has been successfully employed to identify surface active species on metal oxides, such as Co_3_O_4_ and Fe_2_O_3_ (Zandi and Hamann, 2016, Zhang et al., 2014). Compared with the infrared (IR) spectrum of pristine MnO_*x*_-300, four new IR peaks, at 1130, 1036, 980, and 912 cm^−1^, were observed in the spectrum of MnO_*x*_-300 after electrocatalysis at 1.4 V (Figure 2A). The first three peaks are assigned to either or both physisorbed and coordinated phosphate groups, because these peaks are invariable (Figure 2B), potential independent (Figures 2C and S5), and consistent with the IR absorption peaks of potassium phosphates (Figure S6). The absorption peak at 912 cm^−1^ gradually vanishes within 60 min (Figure 2B) and appears only when the applied potential is higher than the water-oxidation onset potential (Figure 2C). We already found that the reduction peak at 0.93 V completely vanished after the first cycle of the negative CV scan (Figures 1B and S2). Consistent with this, no 912 cm^−1^ peak was observed in the IR spectrum of MnO_*x*_-300 after electrocatalysis followed by a negative CV scan (Figure S7). In addition, given the consistency in the degradation rate and the potential-dependent generation between the 912 cm^−1^ peak and the reduction peak at 0.93 V, it is reasonable to attribute the 912 cm^−1^ peak to the same active intermediate state as that observed in the above-mentioned electrochemical study.

To confirm that the observed intermediate state is essentially involved in water oxidation catalyzed by MnO_*x*_-300, we investigated the precursor of MnO_*x*_-300, denoted by MnO_*x*_-as, which has no catalytic activity in water oxidation (Zhang et al., 2017). The IR peak at 912 cm^−1^ was absent from the IR spectrum of MnO_*x*_-as after electrolysis at 1.4 V (Figure 2D). The spectrum showed only one small and invariable peak, at 920 cm^−1^, which can be attributed to phosphate groups, implying that the active intermediate state is not generated on the inactive MnO_*x*_-as. These results confirm the discovery of an active intermediate state with a reduction peak at 0.93 V and IR peak at 912 cm^−1^ for the MnO_*x*_-300-based WOC. The intermediate state is reactive, and its generation strictly corresponds to water-oxidation catalysis, i.e., it is involved in the catalytic cycle.

### The Nature of the Observed Intermediate State

**Figure 3:**
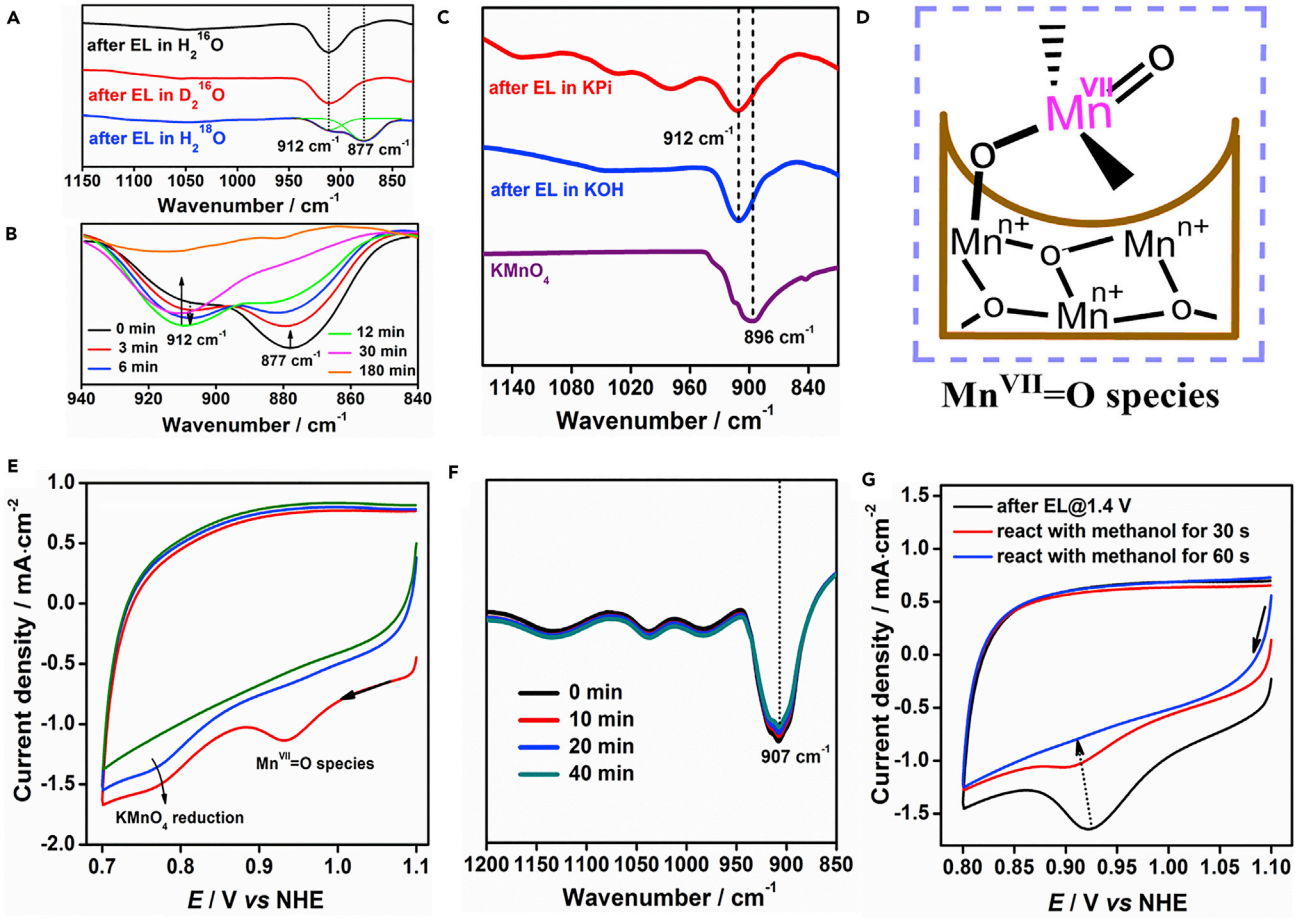
Investigation of the Nature of the Observed Intermediate State and Distinctions between its Reactivity and that of KMnO_4_ (A) IR spectra of MnO_*x*_-300 after electrolysis at 0.85 V in H_2_^16^O, D_2_^16^O, and H_2_^18^O KOH electrolyte. (B) Time-resolved IR spectra of MnO_*x*_-300 after electrolysis at 1.4 V in H_2_^18^O KOH electrolyte. (C) Comparison of IR spectra of KMnO_4_ and MnO_*x*_-300 after electrolysis at 1.4 V in 1.0 M KPi solution and at 0.85 V in 1.0 M KOH electrolyte. (D) Schematic diagram of suggested structure of observed intermediate species. (E) Negative-scan CV curves of MnO_*x*_-300 after electrolysis at 1.4 V in 1.0 M KPi electrolyte with 1 mM KMnO_4_ (the red curve is the first cycle, and the blue curve is the second cycle). The green curve is the second cycle of the CV curve of MnO_*x*_-300 after electrolysis at 1.4 V in 1.0 M KPi electrolyte without KMnO_4_. (F) Time-resolved IR spectra of KMnO_4_ associated with MnO_*x*_-300. (G) Negative-scan CV curves of MnO_*x*_-300 after electrolysis at 1.4 V, followed by reaction with CH_3_OH for different reaction times. See also Figures S8–S15.

Isotopic IR spectroscopic analysis was performed to facilitate assignment of the IR absorption bands of the observed intermediate state. To eliminate overlapping of the IR absorptions of phosphate groups, the catalytic reaction with MnO_*x*_-300 was performed in a KOH solution (see Transparent Methods and Figures S8–S11). The 912 cm^−1^ peak did not shift on substitution of H_2_O with D_2_O in the electrolyte solution, which shows that −OH is not involved in the structure of this intermediate state (Figure 3A). For MnO_*x*_-300 after electrolysis in H_2_^18^O electrolyte, a strong isotopic counterpart at 877 cm^−1^ of the 912 cm^−1^ peak appeared, i.e., an isotopic shift of 35 cm^−1^, suggesting that the surface intermediate species has O in its structure. The time-resolved changes in the multiple isotopic peaks are shown in Figure 3B. In the first 12 min, the intensity of the 877 cm^−1^ peak decreased rapidly and that of the 912 cm^−1^ peak grew significantly. After 30 min, the 877 cm^−1^ peak became indistinct and the 912 cm^−1^ peak intensity decreased compared with that of the peak at 12 min. Both peaks vanished after 180 min. This demonstrates that the O atoms in the intermediates exchange rapidly with either or both atmospheric water and oxygen. This further proves that the intermediate is in a very reactive state.

In previous studies, several species, including Mn^IV^ = O, Mn^V^ = O, Mn–O–O˙, and Mn–O–OH, have been identified in the intermediate states of manganese oxide-based WOCs (Zahran et al., 2016, Najafpour et al., 2016, Jin et al., 2017). However, the vibration frequency of the MnO_*x*_-300 intermediate cannot be attributed to any of these species, because the vibration frequencies of Mn^IV^ = O (Chu et al., 2001, Czernuszewicz et al., 1988), Mn^V^ = O (Chu et al., 2001, Workman et al., 1992), and superoxide-like Mn species (Che and Tench, 1983, Baltanas et al., 1984) generally fall in the region 712–755 cm^−1^, 970–981 cm^−1^, and 1075–1195 cm^−1^, respectively, which are far from the observed vibration frequency, i.e., 912 cm^−1^. Unexpectedly, the 912 cm^−1^ peak closely matches the IR absorption of KMnO_4_, which is at 896 cm^−1^ (Figure 3C). Moreover, an ^18^O isotopic shift of 34 cm^−1^ for KMnO_4_ has been reported (Dong et al., 2002), which is in good agreement with the detected 35 cm^−1^ shift for the intermediates. We observed MnO_4_^−^ release from the surface of the MnO_*x*_ electrode during the first tens of seconds of electrolysis at 1.4 V, but it vanished within a minute (Video S1 and Figure S12). We therefore propose that the active intermediate state consists of a Mn^VII^ = O species bonded to the positively charged surface of MnO_*x*_-300 (Figure 3D).

Video S1. Observation of releasing MnO4− at the beginning period of the catalytic electrolysis, Related to Figure 3 Electrolysis of MnO_*x*_-300 FTO electrode at 1.4 V in 1.0 M KPi pH 7.0 buffer solution.

To clarify the nature of the identified intermediate, we investigated five lines of experimental evidence to distinguish it from free MnO_4_^−^, which is usually a corrosive by-product during water oxidation with Mn catalysts (Limburg et al., 1997, Limburg et al., 1999, Yagi and Narita, 2004). The first two direct differences between the Mn^VII^ = O species and MnO_4_^−^ are the shifts of the CV reduction peak and the IR absorption peak. In addition to the expected reduction of the Mn^VII^ = O species at 0.93 V, another peak, at 0.77 V, which still remained in the second cycle of the negative CV scan, was observed, as shown in Figure 3E. The latter peak clearly corresponds to the reduction of KMnO_4_ in the electrolyte. The higher reduction potential of the Mn^VII^ = O species than that of MnO_4_^−^ shows that the oxidative reactivity of the Mn^VII^ = O species is much higher than that of MnO_4_^−^. The IR peak of the Mn^VII^ = O species is red shifted by 16 nm compared with that of KMnO_4_ (Figure 3C). A similar red shift of the IR peak has been reported for KMnO_4_ adsorbed on β-MnO_2_ (Abbas and Nasser, 2000). This supports the hypothesis that the intermediate species is a Mn^VII^ = O species bound on a MnO_*x*_ nanostructure.

The third argument is the specific degradation of the Mn^VII^ = O species, shown in Figure 2B. To achieve a better comparison with the generated Mn^VII^ = O species, KMnO_4_ was drop-cast on a MnO_*x*_-300 sample after electrolysis at 1.4 V for 20 min and a negative-scan CV was then performed to remove the generated Mn^VII^ = O species. After drying, the mixture was collected for IR spectroscopy. No significant degradation was observed in a mixture of KMnO_4_ and the processed MnO_*x*_-300 (Figure 3F). The observed fast ^18^O exchange of the intermediate in Figure 3B is the fourth solid piece of evidence, because KMnO_4_ does not exchange with ambient oxygen at room temperature (Yiu et al., 2011, Shafirovich et al., 1981). Rapid O exchange with ambient oxygen occurs only for some activated MnO_4_^−^ species, such as [2BF_3_⋅ MnO_4_]^–^ species (Yiu et al., 2011), and a Mn^IV^–Mn^VII^ surface complex (Shafirovich et al., 1981). Direct investigation of the oxidative reactivity of our Mn^VII^ = O intermediate species is the fifth probe (see Transparent Methods and Figures S13–S15). It has been reported that KMnO_4_ can oxidize CH_3_OH at room temperature but the reaction rate is extremely slow, unless the KMnO_4_ is activated by a strong Lewis acid (Du et al., 2011). Here, we found that the degradation of our Mn^VII^ = O species in CH_3_CN with 0.1 M CH_3_OH was complete within 1 min (Figure 3G). In contrast, the degradation of KMnO_4_ under the same conditions was negligible even after 6 hr (Figure S15).

These results fully demonstrate that the identified Mn^VII^ = O species is a disparate species with a reactivity much higher than that of free MnO_4_^−^. The higher reactivity of the Mn^VII^ = O species can be attributed to its bonding to the charged bulk surface, which acts similarly to a Lewis acid, which has been proved to significantly enhance the oxidative reactivity of MnO_4_^−^ (Du et al., 2011, Yiu et al., 2011).

## Discussion

On the basis of these results, we could propose a catalytic water-oxidation mechanism that involves Mn^VII^ = O. However, we first have to address two essential questions.

The first question is what are the rationale and pathway for the formation of Mn^VII^ = O species on *δ*-MnO_*x*_ under such a low potential? In Pourbaix diagrams for Mn, *δ*-MnO_*x*_ borders the MnO_4_^−^ zone (Izgorodin et al., 2012, Najafpour et al., 2016). At pH 7, the theoretical potential for the formation of MnO_4_^−^ is 1.05 V. With an overpotential of 50 mV, it is thermodynamically possible to generate Mn^VII^ species in MnO_*x*_-300 at 1.1 V. Regarding the formation pathway, it has been found that four Mn^IV^ ions can disproportionate into one Mn^VII^ ion and three Mn^III^ ions via a tetranuclear Mn^IV^ intermediate species (Shafirovich et al., 1981, Dzhabiev, 1989). Furthermore, kinetic analysis has shown that two molecules of [(H_2_O) (tpy)Mn(μ-O)_2_Mn(tpy)(H_2_O)] (tpy = 2,2′:6′,2″-terpyridine), i.e., four Mn cores, are involved in the rate-determining step of the formation of MnO_4_^−^ (Yagi and Narita, 2004, Limburg et al., 1999). Strong electronic interactions between Mn sites in MnO_*x*_-300 before the generation of the Mn^VII^ = O sites were also observed in positive scans of MnO_*x*_-300 (see Figure 1A) (Zaharieva et al., 2012). On the basis of these information and our observations, we propose that, in the formation of the Mn^VII^ = O species, multiple Mn sites (both the active Mn site and its surrounding Mn) participate in the process and a complicated disproportionation reaction such as Mn^IV^Mn^IV^Mn^IV^Mn^IV^ to Mn^VII^Mn^III^Mn^III^Mn^III^ may be involved.

The second question is what is the thermodynamic and kinetic feasibility of oxygen evolution from Mn^VII^ = O species? Looking again at the Pourbaix diagrams, *E*(MnO_4_^−^/*δ*-MnO_*x*_) ≈ 1.05 V at pH 7; this is 230 mV higher than the thermodynamic potential for water oxidation. MnO_4_^−^ can therefore spontaneously oxidize H_2_O to O_2_ under neutral conditions via Equation 1 (Skrabal, 1910).

(Equation 1) 4MnO_4_^−^ + 2H_2_O → 4MnO_2_ + 3O_2_ + 4OH^−^

**Figure 4:**
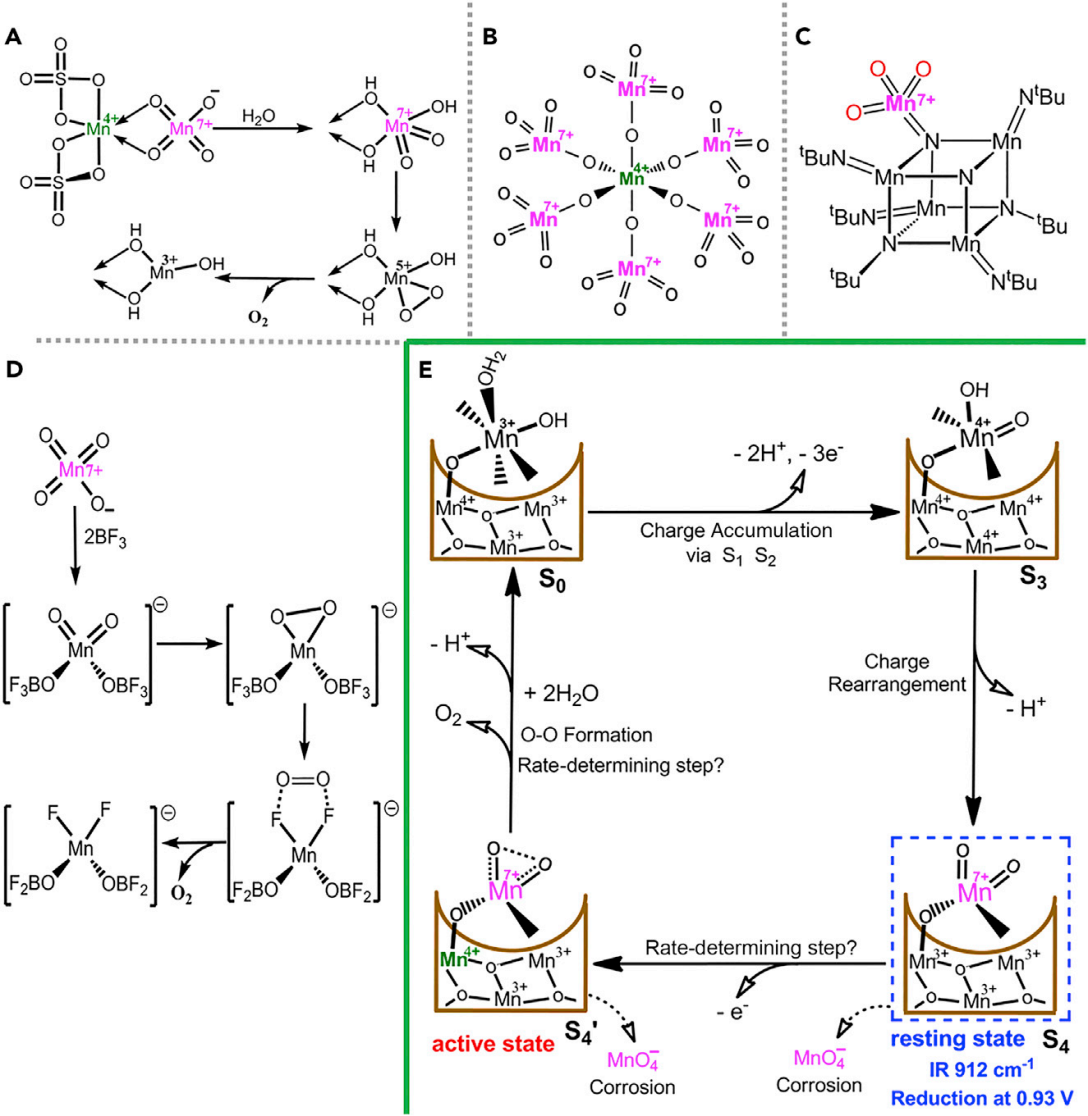
Proposed Catalytic Mechanism Involving Mn^VII^ = O Intermediate for Water Oxidation by MnO_*x*_-300 (A) Pathway for oxygen evolution from MnO_4_^−^ catalyzed by Mn^IV^ molecule (Shafirovich et al., 1981). (B) Structure of the [Mn^IV^(Mn^VII^O_4_)_6_]^2−^ complex (Krebs and Hasse, 1974). It rapidly produces O_2_ at T ≥ −4°C. (C) Structure of the cubic Mn–nitride complex (Vaddypally et al., 2017). Pendant Mn^VII^ = O moiety is preferentially reactive in comparison with free MnO_4_^−^. (D) Mechanism for fast oxygen evolution from MnO_4_^−^ with activation by a strong Lewis acid, i.e., BF_3_ (Yiu et al., 2011). Oxygen evolution from 7.6 mM KMnO_4_ completed within 200 s with the activation of 0.12 mM BF_3_⋅CH_3_CN. (E) Proposed catalytic cycle, involving Mn^VII^ = O, in MnO_*x*_-300-catalyzed water-oxidation reaction. The overall mechanistic process involves charge accumulation (S_0_ → S_3_), charge rearrangement (S_3_ → S_4_), active-state formation (S_4_ → S_4_′), and oxygen evolution (S_4_′ → S_0_).

Although the rate of oxygen evolution from free MnO_4_^−^ is very slow, it has been reported that the rate increases significantly if MnO_4_^−^ is bonded with MnO_2_, a Mn^4+^ ion, or a Lewis acid (Skrabal, 1910, Yiu et al., 2011, Shafirovich et al., 1981). It has been known since 1910 that MnO_2_ catalyzes the reaction of MnO_4_^−^ with H_2_O (Skrabal, 1910). Shafirovich et al. studied the kinetics of the reaction of MnO_4_^−^ with H_2_O, with Mn^4+^ as a catalyst. They suggested that a Mn^IV^–Mn^VII^ surface complex is the key intermediate for O–O formation in the catalytic mechanism (Figure 4A) (Shafirovich et al., 1981, Shafirovich, 1978). The crystal structure of a (H_3_O)_2_–[Mn^IV^(Mn^VII^O_4_)_6_]⋅11H_2_O intermediate complex, which rapidly evolves oxygen at *T* ≥ −4°C, has been identified by Krebs and Hasse (Figure 4B) (Krebs and Hasse, 1974). Recently, a highly active pendant Mn^VII^ = O moiety on a cubic Mn–nitride complex was suggested as a synthetic structural model of the proposed S4 state in PSII (Figure 4C) (Vaddypally et al., 2017). The work by Lau's group shows that MnO_4_^−^ activated by a strong Lewis acid, namely, BF_3_, rapidly evolves O_2_ via intramolecular coupling of two Mn–oxo species (Figure 4D) (Yiu et al., 2011). A Mn^VII^–nitrido complex was also reported by the same group as an essential intermediate in Ce^IV^-driven water oxidation (Ma et al., 2015). All these previous reports suggest that oxygen evolution at a Mn^VII^ = O site bonded to an oxidized cluster is not only thermodynamically possible but also has rapid kinetics.

Finally, we propose a catalytic cycle that involves Mn^VII^ = O for water-oxidation catalysis by MnO_*x*_-300 (Figure 4E). One active Mn site and three related Mn atoms are assumed to participate actively in the catalysis. After multiple charge accumulation accompanied by transfer of three electrons and two protons, the initial state, i.e., [Mn^III^Mn^III^Mn^IV^(HO−Mn^III^−OH_2_)] (S_0_), is oxidized to [Mn^IV^Mn^IV^Mn^IV^(HO–Mn^IV^ = O)] (S_3_) via states S_1_ and S_2_. State S_3_ is assumed to be a transition state, which will undergo charge rearrangement with the release of one proton, resulting in a resting state, [Mn^III^Mn^III^Mn^III^Mn^VII^(=O)_2_] (S_4_), which probably contains a dangling Mn^VII^ = O site. The observed CV reduction peak at 0.93 V and the IR absorption frequency of 912 cm^−1^ can be related to this Mn^VII^ = O site in S_4_. Before O–O bond formation, the Mn atom directly bonded to the dangling Mn^VII^ = O site is thought to be further oxidized to Mn^IV^, which acts as a strong Lewis acid and promotes the reactivity of the Mn^VII^ = O site, forming the active state [Mn^III^Mn^III^Mn^IV^Mn^VII^(=O)_2_] (S_4_′). Along with oxygen evolution from S_4_′, the involved Mn cluster returns to its starting state S_0_ by binding two H_2_O molecules to the empty sites and losing one proton, completing one catalytic cycle. Finally, we would like to point out that the commonly observed essential Mn^III^ species is probably formed in the charge rearrangement step and the corrosive product MnO_4_^−^ is probably formed by the detachment of the Mn^VII^ = O site from either or both S_4_ and S_4_′, because the Mn^IV^−O bond in the Mn^IV^−O−Mn^VII^ = O moiety should be a weak bond.

This proposed mechanism with an ultrahigh-valent intermediate is not unprecedented. A similar mechanism involving an ultrahigh-valent Ru^VIII^O_4_ intermediate species has been reported for RuO_*x*_ catalysts (Giordano et al., 2016). Recently, coupling of Fe^VI^-peroxo is also proposed as the O−O formation mechanism for the Ni-Fe layered double hydroxide catalyst (Hunter et al., 2018). Our proposed mechanism is consistent with the facts that the essential species Mn^III^ is always present in active Mn catalysts and that MnO_4_^−^ is often observed in catalyst corrosion. Accordingly, we believe that, on the basis of our extensive investigations of the Mn^VII^ = O intermediate in this study, the proposed mechanism involving Mn^VII^ = O is highly probable and reliable. It offers cogent guidance for developing more efficient synthetic WOCs, and it might also be valid for the Mn_4_CaO_5_ cluster in PSII.

## Methods

All methods can be found in the accompanying Transparent Methods supplemental file.

## Supplemental Information

Document S1. Transparent Methods and Figures S1–S15
