# Supplementary material for: Identifying MnVII-oxo Species during Electrochemical Water Oxidation by Manganese Oxide
Source: iScience. 2018 May 30;4:144–52. doi: 10.1016/j.isci.2018.05.018 (PMC6147022; doi:10.1016/j.isci.2018.05.018)
Supplement: Document S1. Transparent Methods and Figures S1–S15 [file mmc1.pdf]

ISCI, Volume *4*

Supplemental Information

Identifying Mn^{VII}-oxo Species during Electrochemical Water Oxidation by Manganese Oxide

Biaobiao Zhang, Quentin Daniel, Lizhou Fan, Tianqi Liu, Qijun Meng, and Licheng Sun

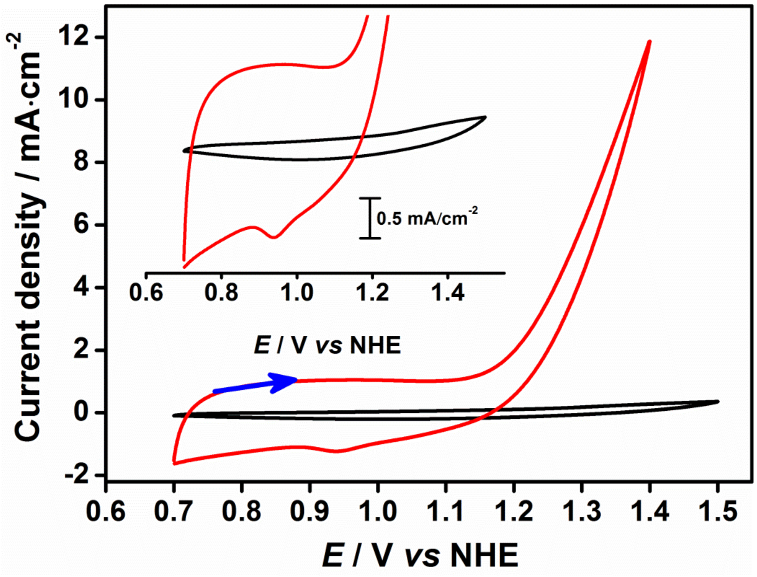

Figure S1. Comparison of CV curves of MnO_x-300 in aqueous and CH_2Cl_2 electrolyte. Related to Figure 1. CV curves of MnO_x-300 in 1.0 M KPi electrolyte (red line) and in CH_2Cl_2 with 0.1 M *n*-Bu_4NPF_6 electrolyte (black line). Scan rate 10 mV/s. The inset shows the enlarged parts of the CV curves.

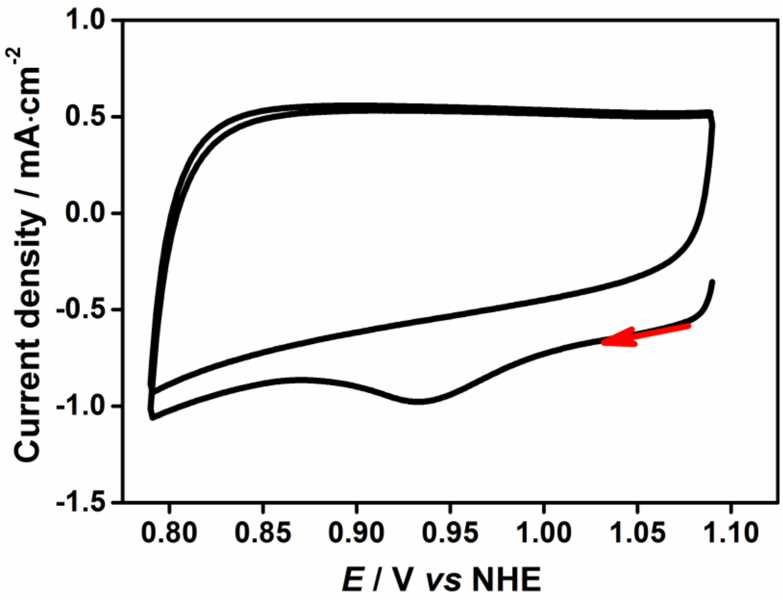

Figure S2. Electrochemical investigation on the generation of the intermediate during water oxidation. Related to Figure 1. Negative-scanned CV curves of MnO_x-300 after electrolysis at 1.4 V for 2 min. 1.0 M KPi electrolyte. Scan rate 10 mV/s.

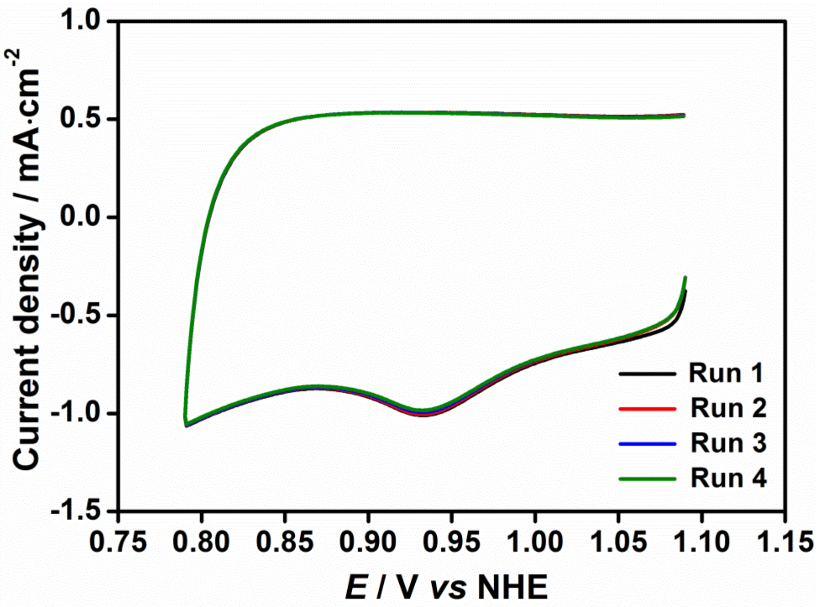

Figure S3. Electrochemical investigation on the generation of the intermediate during water oxidation. Related to Figure 1. Four repeats of negative-scanned CV curves of MnO_x-300 after electrolysis at 1.4 V for 2 min. 1.0 M KPi electrolyte. Scan rate 10 mV/s.

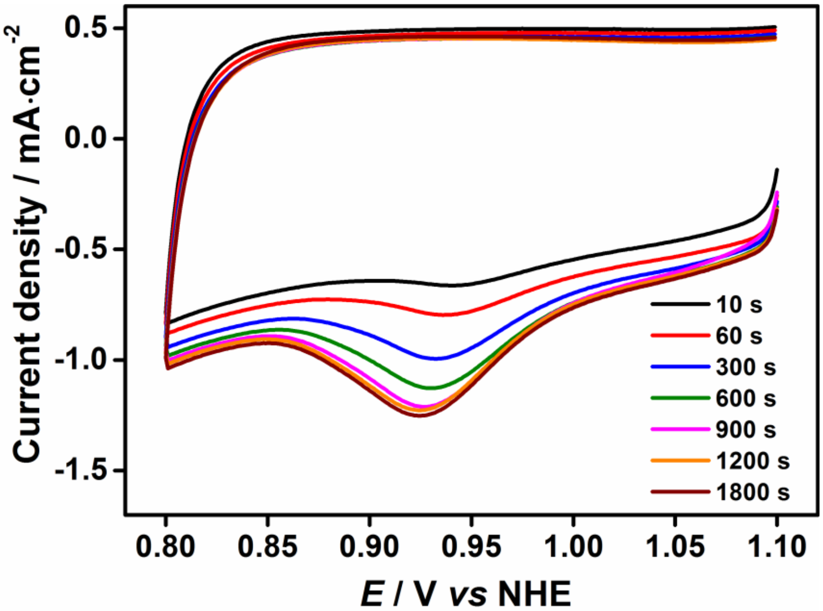

Figure S4. Electrochemical investigation on the generation of the intermediate during water oxidation. Related to Figure 1. Negative-scanned CV curves of MnO_x-300 after electrolysis at 1.4 V for different delay times. 1.0 M KPi electrolyte. Scan rate 10 mV/s.

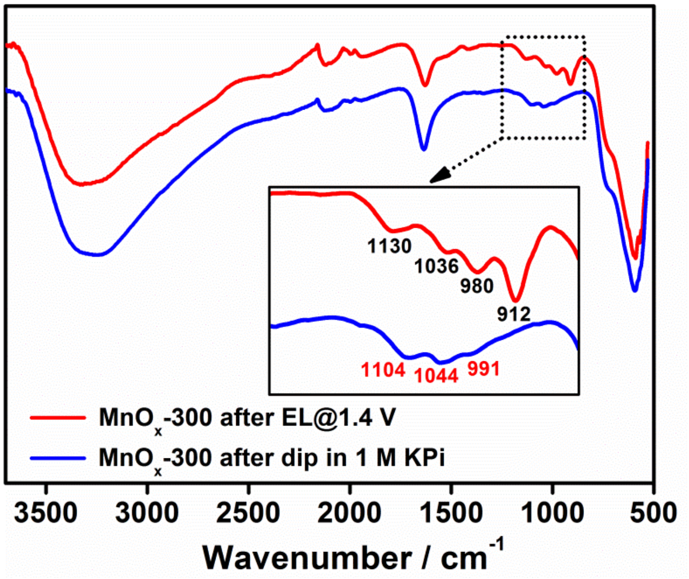

Figure S5. Assignment of the former three IR peaks at 1130 cm^{-1}, 1036 cm^{-1}, 980 cm^{-1}. Related to Figure 2. IR spectra of MnO_x-300 after electrolysis at 1.4 V and MnO_x-300 after dipping in 1.0 M KPi solution. The peaks at 1130 cm^{-1}, 1036 cm^{-1}, 980 cm^{-1} are observed for the MnO_x-300 after dipping in KPi electrolyte. However, the 912 cm^{-1} peak is completely absent.

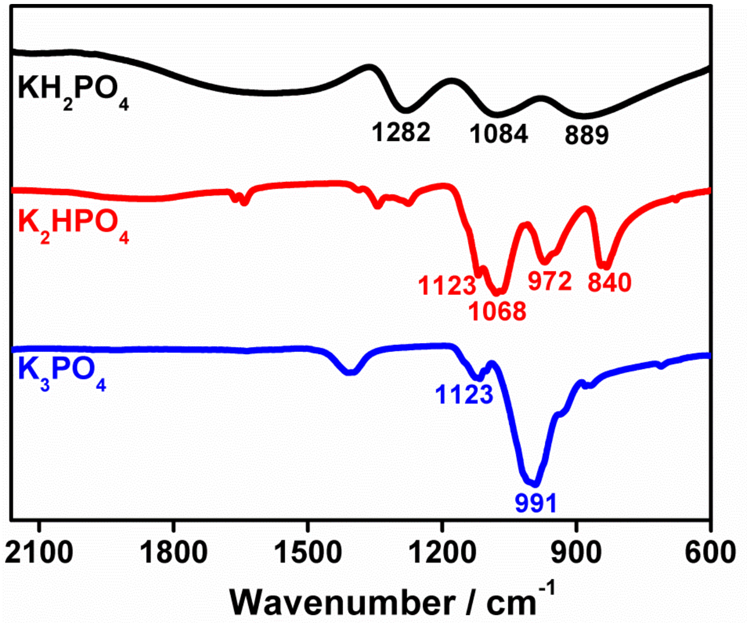

Figure S6. Assignment of the former three IR peaks at 1130 cm^{-1}, 1036 cm^{-1}, 980 cm^{-1}. Related to Figure 2. IR spectra of K_3PO_4, K_2HPO_4 and KH_2PO_4 salts. The peaks at 1130 cm^{-1}, 1036 cm^{-1}, 980 cm^{-1} are all covered in the absorption bands of phosphates.

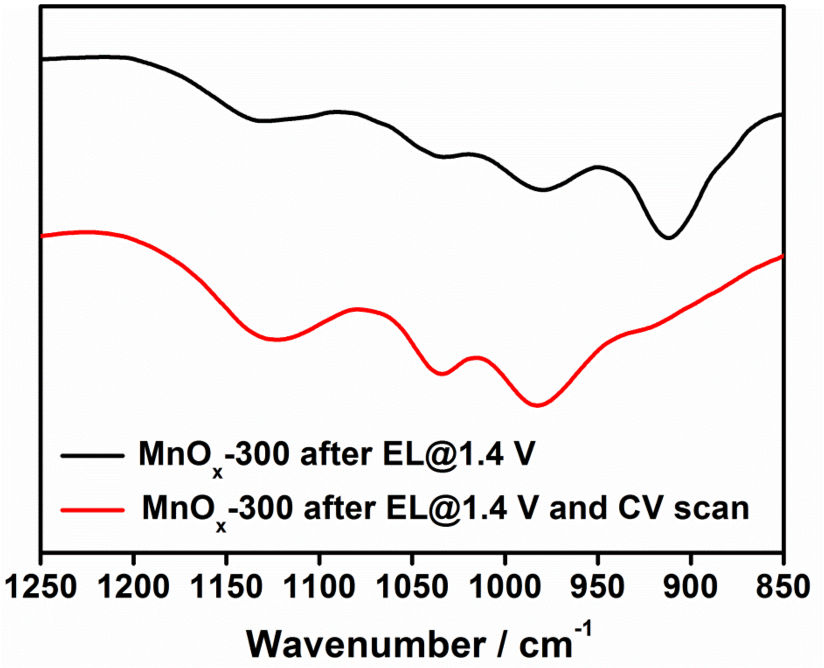

Figure S7. Related to Figure 2. IR spectra of MnO_x-300 after electrolysis at 1.4 V and MnO_x-300 after electrolysis at 1.4 V followed by negative CV scan.

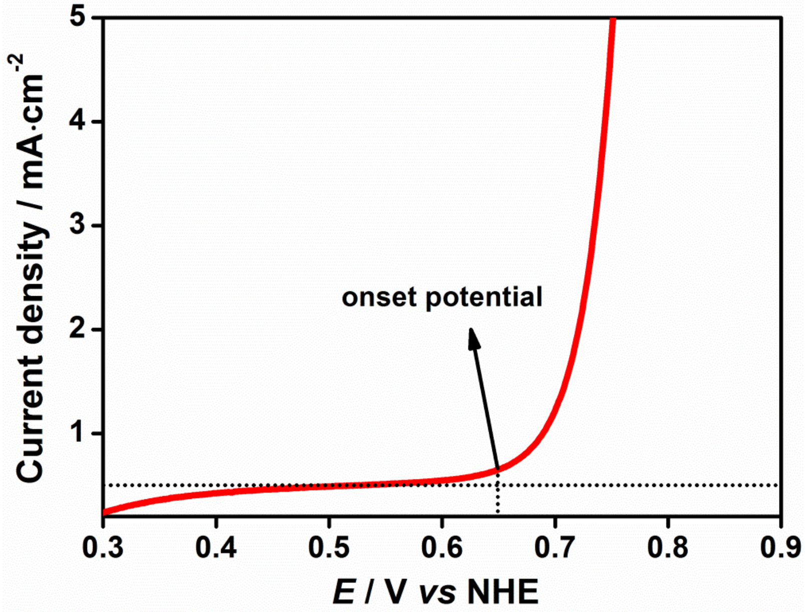

Figure S8. Related to Figure 3. LSV curves of MnO_x-300 in 1.0 M KOH. Scan rate 10 mV/s.

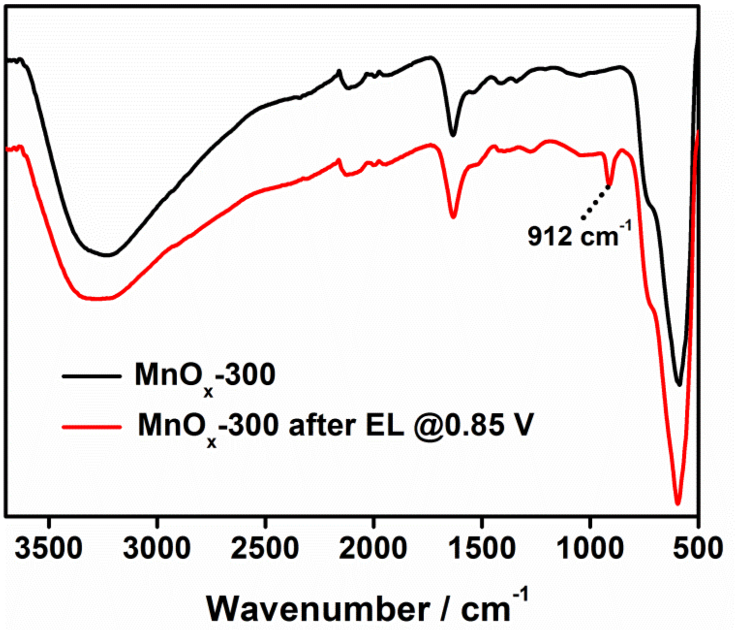

Figure S9. Related to Figure 3. IR spectra of pristine MnO_x-300 and MnO_x-300 after electrolysis at 0.85 V in 1.0 M KOH electrolyte.

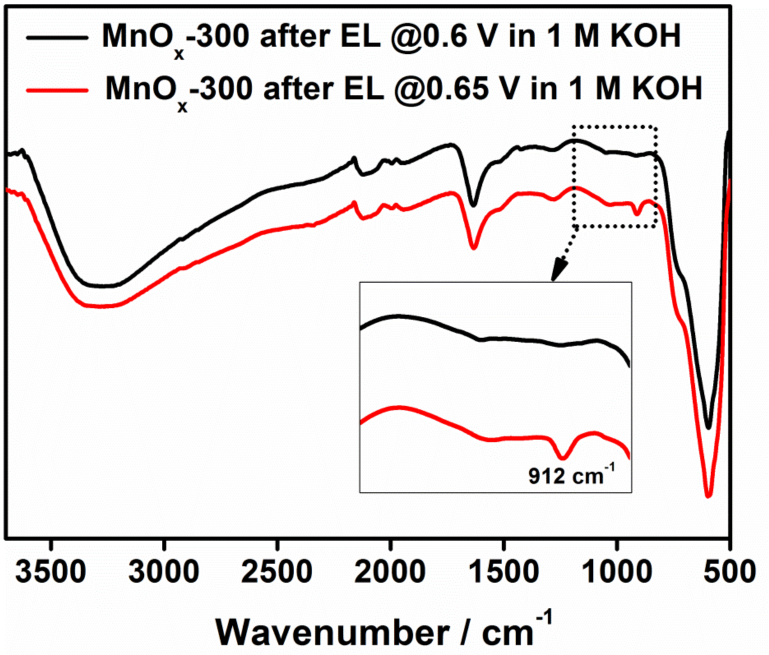

Figure S10. Related to Figure 3. IR spectra of MnO_x-300 after electrolysis at 0.6 V and 0.65 V in 1.0 M KOH electrolyte.

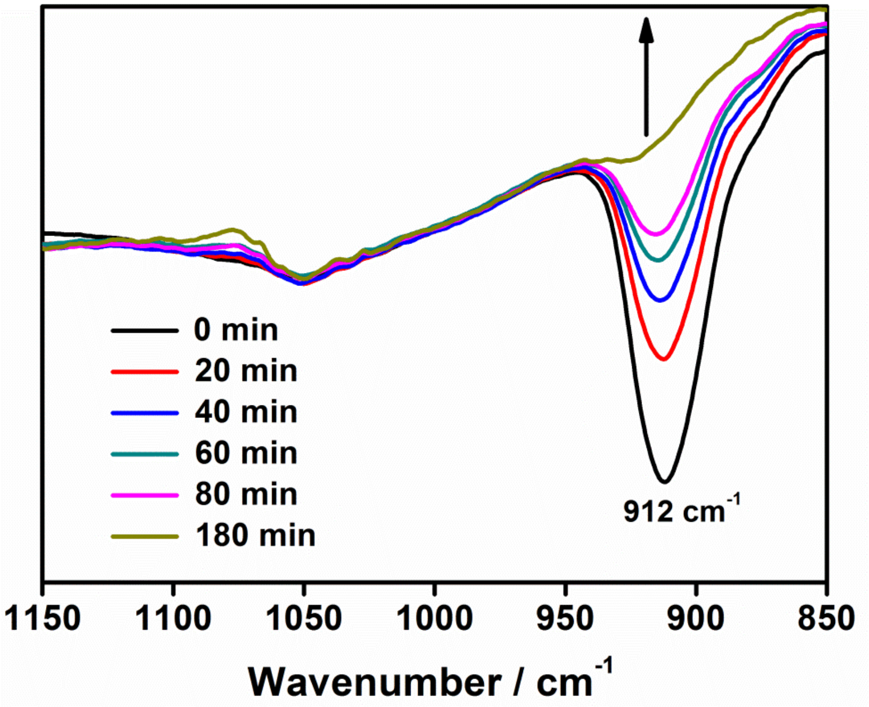

Figure S11. Related to Figure 3. Time-resolved IR spectra of MnO_x-300 after electrolysis 0.85 V in 1.0 M KOH electrolyte.

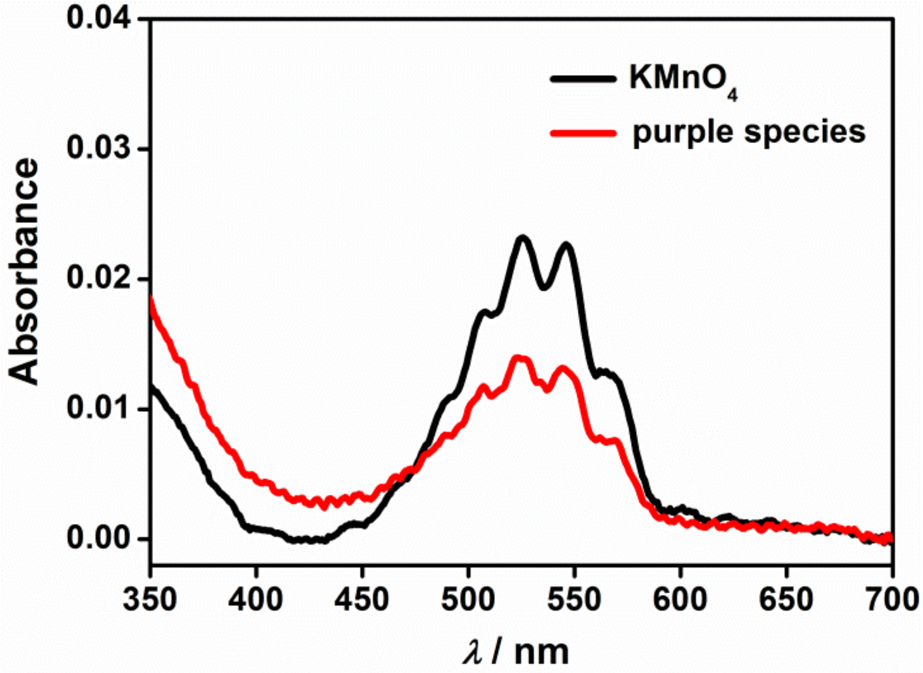

Figure S12. Related to Figure 3. UV-Vis spectra of $KMnO_4$ and the solution of the purple species from the MnO_x-300 electrode at the very first tens of seconds of electrolysis at 1.4 V.

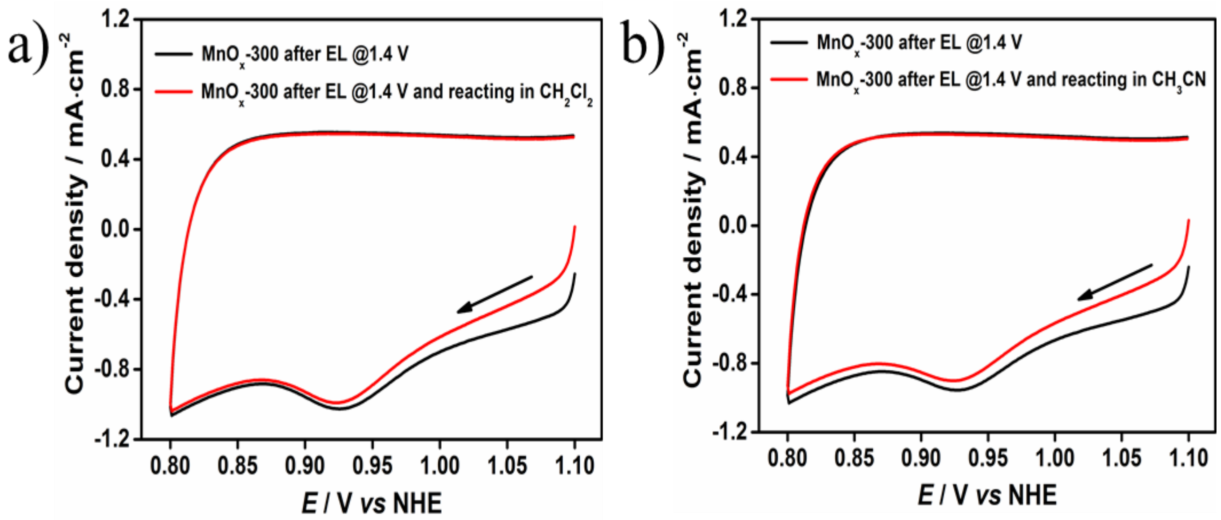

Figure S13. Related to Figure 3. Negative-scanned CV curves of MnO_x-300 after electrolysis at 1.4 V followed by reaction for 1.0 min with a) CH_2Cl_2; b) CH_3CN. 1.0 M KPi electrolyte. Scan rate 10 mV/s.

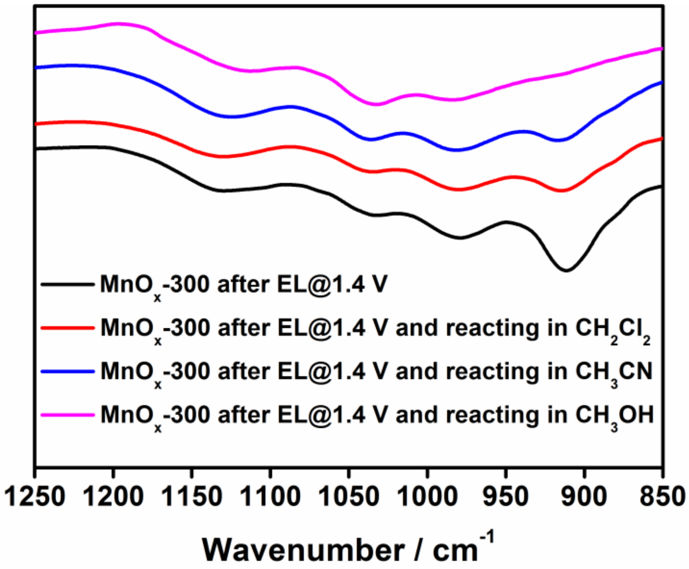

Figure S14. Related to Figure 3. IR spectra of MnO_x-300 after electrolysis at 1.4 V in 1.0 M KPi followed by reaction for 1.0 min with CH_3OH and for 5.0 min with CH_2Cl_2 and CH_3CN.

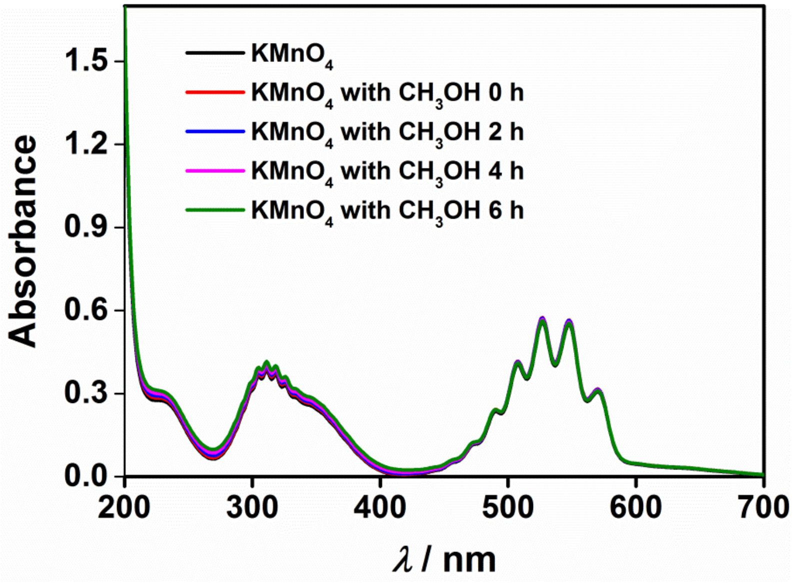

Figure S15. Related to Figure 3. UV-Vis spectra of 0.1 mM $KMnO_4$ and 0.1 mM $KMnO_4$ with 0.1 M methanol for 0 h, 2 h, 4 h and 6 h.

Transparent Methods

Materials

$Mn(CH_3COO)_2 \cdot 4H_2O$, NaCl, KH_2PO_4, K_2HPO_4, K_3PO_4, KOH, $KMnO_4$, CH_3CN, CH_3OH, CH_2Cl_2, D_2O (99.9 atom % D), 40 wt. % KOD in D_2O (98 atom % D) and $H_2{}^{18}O$ (97 atom % ^{18}O) were purchased from Sigma-Aldrich. All solvents and reagents are used as received. Ultra pure water (18.2 $M\Omega\cdot cm^{-1}$) for all the reactions or measurements was obtained from a Milli-Q system. Fluorine-doped tin oxide (FTO) substrates were purchased from Pilkington (resistance of ~8 $\Omega\cdot cm^{-2}$) and were cleaned inside an ultrasonic bath in water and ethanol for 30 min.

Preparation of MnO_x-300

The precusor manganese oxide film MnO_x-as was electro-deposited from an aqueous solution of 5 mM $(CH_3COO)_2Mn$ and 50 mM NaCl at 1.4 V for 30 minutes. Further annealing at 300 ℃ for 2 hours was performed to obtain the active MnO_x-300.

Electrochemical Measurements

All electrochemical experiments employed a CHI 650e potentiostat in a single-compartment cell with a three-electrode configuration. The cell was equipped with a FTO electrode with manganese oxide film as the working electrode, a platinum mesh as the counter electrode and an Ag/AgCl (3.5 M KCl in water) as the reference electrode. All experiments were conducted at ambient temperature (~25 ℃). All cyclic voltammograms (CV) and linear scan voltammograms (LSV) were taken with scan rate of 10 mV s^{-1}. Potential versus NHE was calibrated by using $Ru(bpy)_3Cl_2$ as a reference with $E(Ru^{II/III})$ = 1.26 V.

Infrared Spectroscopy Measurements

The attenuated total reflectance Fourier-transform infrared spectroscopy (ATR-FTIR) spectra were measured on a Thermo Scientific Nicole Is5 FT-IR spectrometer. For the general ATR-FTIR determination, manganese oxide electrodes treated after various processes were carefully rinsed by pure water and dried quickly by a N_2 flow for tens of seconds; then the manganese oxides were quickly scraped from the FTO electrode to determine their infrared absorption spectra.

Comparison of CV curves of MnO_x-300 in aqueous and CH_2Cl_2 electrolyte

When a CH_2Cl_2 solution with 0.1 M *n*-Bu_4NPF_6 was used in place of the aqueous electrolyte which is indispensable for the water oxidation reaction, there was no distinct reduction peak in the CV curve of MnO_x-300 within the potential range of 0.7-1.5 V (Figure S1).

Electrochemical investigation on the generation of the intermediate during water oxidation

After the MnO_x-300 was electrolyzed at 1.4 V for 2 min, where the catalytic water oxidation was fast, the following negative-scanned CV showed in the first cycle a large reduction peak, indicating abundance of the intermediates were formed and accumulated in MnO_x-300 during water oxidation (Figure S2). The reduction peak completely disappeared in the second cycle because the generated intermediates have been reduced in the first scanning. Repeats of the above process showed coincident CV curves. It means that the generation of the intermediate after the same process of electrolysis is repeatable (Figure S3). We also found that the formation of the intermediate needs at least 15 min to reach a saturation value along with the equilibrium of electrocatalytic water-oxidation reaction (Figure S4).

Consistency between the IR peak at 912 cm^{-1} and CV reduction peak at 0.93 V

It shows in the previous section that the CV peak of the intermediate completely vanishes after the first cycle of the negative CV scan (Figure S2). Consistently, there is no 912 cm^{-1} peak in the IR spectra of electrolyzed MnO_x-300 after negative CV scan (Figure S7). The consistency between the transient IR peak at 912 cm^{-1} and CV reduction peak at 0.93 V is further explored in the Supplementary Section 8, where the presence or absence of the IR peak at 912 cm^{-1} and the reduction peak at 0.93 V is consistent with each other and corresponds to the maintenance or degradation of the generated intermediate after reaction with different organics, respectively.

Isotopic IR spectroscopy

In order to get rid of the overlap in the IR absorption (e.g. band 1036 cm^{-1} and 980 cm^{-1}) of the phosphate group, the related catalysis by MnO_x-300 was carried out by employing 1.0 M KOH solution, in which the water-oxidation onset potential of MnO_x-300 is 0.65 V (Figure S8). In the IR spectrum of MnO_x-300 after electrolysis at 0.85 V where the rate of water oxidation is fast, 912 cm^{-1} is the only distinct new peak compared to the IR spectrum of pristine MnO_x-300

(Figure S9). The same as under neutral conditions, the 912 cm^{-1} peak only shows up when the applied potential of electrolysis is higher than onset potential of 0.65 V (Figure S10). The slow degradation of the 912 cm^{-1} peak was also observed (Figure S11). These indicate that the intermediate species involved in the catalysis under basic conditions is the same as the one revealed under neutral conditions. Therefore, it is rational to perform the isotopic IR spectroscopic study with the catalysis in KOH electrolyte solution.

1.0 M KOD-D_2O electrolyte was prepared from D_2O (99.9 atom % D) and 40 wt. % KOD in D_2O (98 atom % D). 1.0 M KOH-$H_2^{18}O$ electrolyte was prepared from $H_2^{18}O$ (97 atom % ^{18}O) and KOH. The final ratio of ^{18}O-labelled oxygen in the electrolyte is about 93%. MnO_x-300 was electrolyzed at 0.85 V in either 1.0 M KOD-D_2O electrolyte or 1.0 M KOH-$H_2^{18}O$ electrolyte. After electrolysis, the MnO_x-300 was carefully rinsed by either D_2O (99.9 atom % D) or $H_2^{18}O$ (97 atom % ^{18}O), respectively. The MnO_x-300 was then quickly dried by N_2 flow for tens of seconds. The manganese oxide was scraped off and collected for its IR spectrum.

Assignment of the 912 cm^{-1} peak

The 912 cm^{-1} peak matches with the IR absorption frequencies of $KMnO_4$, which is at 896 cm^{-1} (Figure 3c). Moreover, we did observe some purple colored species releasing from the surface of the MnO_x electrode during the very first tens of seconds of the electrolysis at 1.4 V, and then vanished in an minute (see Supplemental Video 1). The solution with the purple colored species was quickly taken out from the electrode surface and detected by UV-Vis, which clearly identified it as MnO_4^- (Figure S12). These observations prove that Mn^{VII} can be reached during water-oxidation catalysis.

Reactivity of the Mn^{VII}=O intermediate

Reactivity of the Mn^{VII}=O species on the electrolyzed MnO_x-300 was studied by reacting it with 0.1 M CH_3OH in CH_3CN. The MnO_x-300 after electrolysis at 1.4 V in KPi electrolyte was carefully rinsed by water and quickly dried by N_2 flow. Then this processed MnO_x-300 was dipped into a solution of 0.1 M CH_3OH in CH_3CN to let the generated Mn^{VII}=O species react with CH_3OH. After different reaction times, the consumptions of the Mn^{VII}=O species were determined by their negative-scanned CVs. Results show that the Mn^{VII}=O species completely vanished in one minute (Figure 3g). In the control experiments, plenty of the Mn^{VII}=O species

still remained after reaction for one minute, when the stable organics, such as pure CH_2Cl_2 and CH_3CN, were used instead of 0.1 M CH_3OH as substrate (Figure S13). The degradation and sustainment of the Mn^{VII}=O species can also be determined by its IR absorption at 912 cm^{-1} (Figure S14).

The reaction of 0.1 mM $KMnO_4$ with 0.1 M CH_3OH in CH_3CN was monitored by UV-Vis absorption, which was measured with a PerkinElmer Lambda 750 UV-Vis spectrophotometer. After even reaction for 6 h, the degradation of the $KMnO_4$ is still negligible. (Figure S15). These results support that the Mn^{VII}=O species is much more reactive than MnO_4^-.
